# Functional dissection of the developmentally restricted BEN domain chromatin boundary factor Insensitive

**DOI:** 10.1186/s13072-018-0249-2

**Published:** 2019-01-03

**Authors:** Anna Fedotova, Chaevia Clendinen, Artem Bonchuk, Vladic Mogila, Tsutomu Aoki, Pavel Georgiev, Paul Schedl

**Affiliations:** 1 Department of Genetics, Institute of Gene Biology, Russian Academy of Sciences, Moscow, Russia; 2 Department of Molecular Biology, Princeton University, Princeton, NJ USA

## Abstract

**Background:**

Boundaries in the *Drosophila* bithorax complex delimit autonomous regulatory domains that activate the parasegment (PS)-specific expression of homeotic genes. The *Fab*-*7* boundary separates the *iab*-*6* and *iab*-*7* regulatory domains that control *Abd*-*B* expression in PS11 and PS12. This boundary is composed of multiple functionally redundant elements and has two key activities: it blocks crosstalk between *iab*-*6* and *iab*-*7* and facilitates boundary bypass.

**Results:**

Here, we have used a structure–function approach to elucidate the biochemical properties and the in vivo activities of a conserved BEN domain protein, Insensitive, that is associated with *Fab*-*7*. Our biochemical studies indicate that in addition to the C-terminal BEN DNA-binding domain, Insv has two domains that mediate multimerization: one is a coiled-coil domain in the N-terminus, and the other is next to the BEN domain. These multimerization domains enable Insv to bind simultaneously to two canonical 8-bp recognition motifs, as well as to a ~ 100-bp non-canonical recognition sequence. They also mediate the assembly of higher-order multimers in the presence of DNA. Transgenic proteins lacking the N-terminal coiled-coil domain are compromised for boundary function in vivo. We also show that Insv interacts directly with CP190, a protein previously implicated in the boundary functions of several DNA-binding proteins, including Su(Hw) and dCTCF. While CP190 interaction is required for Insv binding to a subset of sites on polytene chromosomes, it has only a minor role in the boundary activity of Insv in the context of *Fab*-*7*.

**Conclusions:**

The subdivision of eukaryotic chromosomes into discrete topological domains depends upon the pairing of boundary elements. In flies, pairing interactions are specific and typically orientation dependent. They occur in *cis* between neighboring heterologous boundaries, and in *trans* between homologous boundaries. One potential mechanism for ensuring pairing-interaction specificity is the use of sequence-specific DNA-binding proteins that can bind simultaneously with two or more recognition sequences. Our studies indicate that Insv can assemble into a multivalent DNA-binding complex and that the N-terminal Insv multimerization domain is critical for boundary function.

**Electronic supplementary material:**

The online version of this article (10.1186/s13072-018-0249-2) contains supplementary material, which is available to authorized users.

## Background

Gene regulation in multicellular eukaryotes depends upon special elements called chromatin boundaries or insulators. These elements coordinate regulatory interactions with chromosome architecture by subdividing the chromosome into a series of functionally autonomous looped domains [1–4]. The subdivision of the chromosome into looped domains in flies depends upon boundary–boundary pairing interactions [1, 5–8]. These pairing interactions are highly specific and typically orientation dependent. Pairing between neighboring boundaries in *cis* can be head-to-head or head-to-tail, and these pairing configurations are predicted to give rise to loops with quite different topologies [6, 9–11]. Fly boundaries also pair with themselves in *trans* [6, 12]. Self-pairing interactions align and pair homologues in register, and this facilitates *trans*-regulatory interactions or transvection. Unlike heterologous boundary pairing interactions, self-pairing interactions appear to be exclusively head-to-head. Both specificity and orientation dependence in pairing interactions are determined by the proteins associated with each element. While the only DNA-binding protein linked to boundary elements in vertebrates is the polydactyl C2H2 zinc finger protein CTCF, more than a dozen DNA-binding proteins have been implicated in boundary function in flies [7, 13–15]. In addition to the fly CTCF protein, several other fly polydactyl C2H2 zinc finger proteins are known to have insulator functions including Su(Hw), Pita, Zw5, Zipic, and Opbp [15–18].

Other widely conserved DNA-binding protein families also have insulator functions in flies. One of these is the family of BEN DNA-binding domain proteins [19, 20]. Vertebrate genomes encode multiple BEN domain proteins, while *Drosophilids* have four closely related BEN domain proteins: Insensitive (Insv), Elba1, Elba2, and Mod (mdg4) [19–21]. Unlike most of the known polydactyl C2H2 zinc finger proteins, the boundary functions of these three BEN domain proteins are developmentally restricted. Insv is ubiquitously expressed during early embryogenesis; however, after gastrulation, its expression becomes progressively restricted to a subset of cells in the CNS and PNS [22, 23]. The BEN domain proteins Elba1 and Elba2 are part of a tripartite complex that also contains a linker protein Elba3. The three Elba proteins are expressed during the midblastula transition; however, their expression shuts off after the formation of the cellular blastoderm [21, 24, 25]. Genome-wide chromatin immunoprecipitation experiments (ChIPs) have shown that Insv co-localizes with several known boundary proteins including CTCF, Mod(mdg4), BEAF, and CP190 at relatively high frequencies [21]. Moreover, in the case of CP190, this co-localization is not coincidental as these two proteins were found to co-immunoprecipitate [21].

**Fig. 1:**
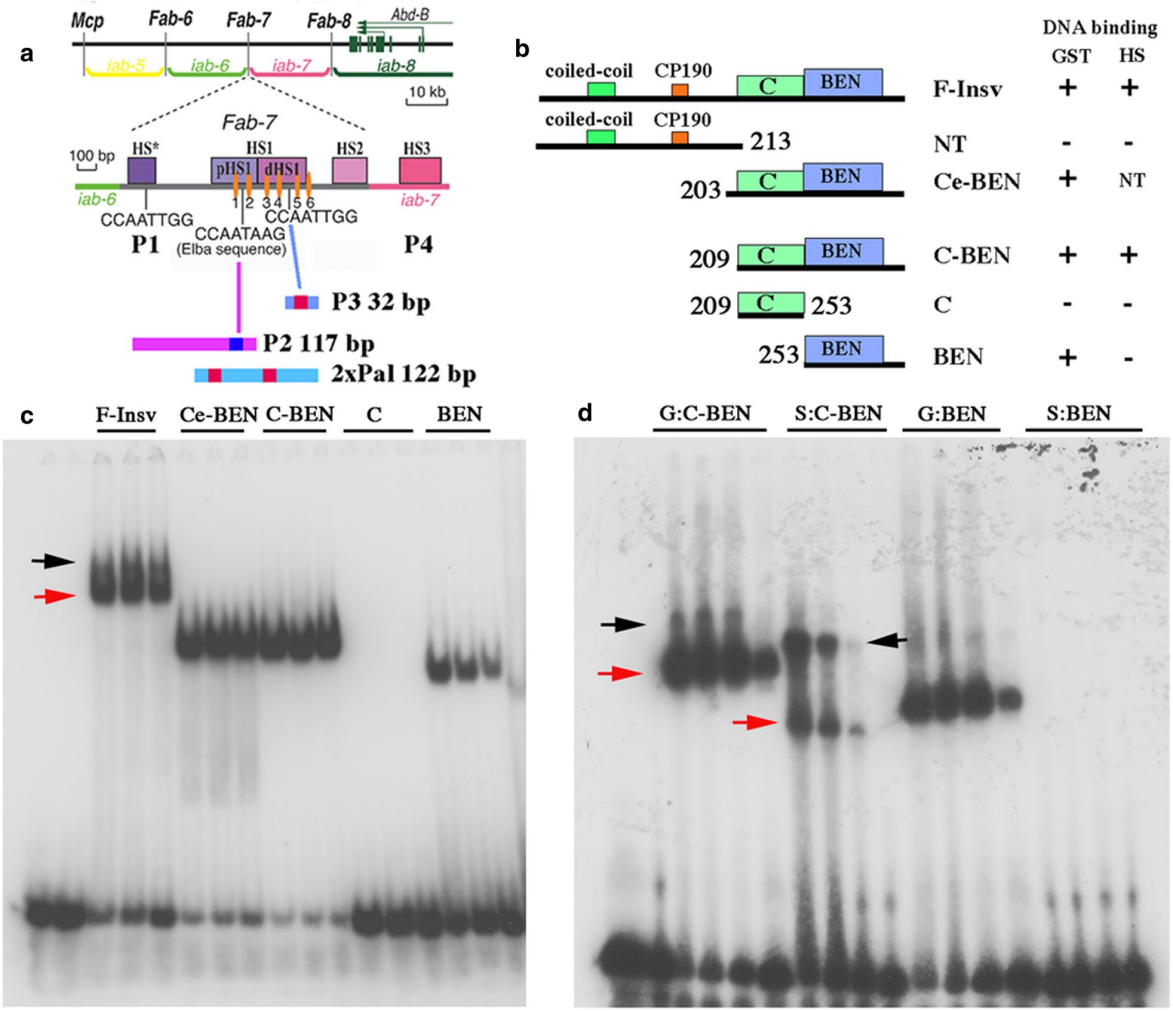
Organization of the bithorax complex and functional dissection of the Insv protein: DNA binding and multimerization. **a** Map of the *Abd*-*B* region of the bithorax complex. The relative location of the *Abd*-*B* regulatory domains, *iab*-*5*, *iab*-*6*, *iab*-*7,* and *iab*-*8*, and the positions of the boundary elements, *Mcp*, *Fab*-*6*, *Fab*-*7,* and *Fab*-*8,* and the *Abd*-*B* transcription unit. Map of the *Fab*-*7* nuclease hypersensitive sites, HS*, HS1, HS2, and HS3 (*iab*-*7* PRE), and location of the binding sites for the GAGA factor and Insv. Probes for EMSA experiments P3, P2, and 2xPal. P3 is a 32-bp sequence derived from distal side of HS1 (dHS1). Position of the CCAATTGG palindrome is indicated by red box. P2 is a 117-bp sequence derived from the proximal side of HS1. On the right end it contains the Elba sequence CCAATAAG (blue box) and a binding site for the GAGA factor (orange oval). 2xPal is a 122-bp artificial sequence containing two CCAATTGG palindromes. **b** Full-length and various truncated versions of the Insv protein are expressed in bacteria and then tested for DNA-binding activity. Schematic diagram of the Insv proteins tested for DNA-binding activity. The bacterially expressed proteins were tagged with either GST (G) or His-Sumo (HS). DNA-binding activity is indicated by (+) or (−). **c** EMSA of probe P3 by GST-tagged full-length and truncated Insv proteins. **d** EMSA of C-BEN and BEN proteins (see diagram in **b**) tagged with GST (G) or His-Sumo (HS). Amount of protein added (left to right) to the reaction mix was estimated based on the relative intensity of the Coomassie-stained protein bands in SDS-PAGE gels: 0.169 μm, 0.085 μm, and 0.042 μm

While genome-wide ChIPs for the Elba proteins have not yet been published, both the Elba complex and Insv have been implicated in the functioning of the bithorax complex (BX-C) *Fab*-*7* boundary [21, 24, 25]. BX-C contains three homeotic genes: *Ultrabithorax* (*Ubx*), *abdominal*-*A* (*abd*-*A*), and *Abdominal*-*B* (*Abd*-*B*). These three genes specify the identity of cells in the parasegments (PS5-13) that make up the posterior two-thirds of the fly [26, 27]. Expression of the three homeotic genes is controlled by an ~ 300-kb regulatory region that is subdivided into a series of autonomous parasegment (PS)-specific regulatory domains. For example, the *iab*-*5, iab*-*6, iab*-*7,* and *iab*-*8 cis*-regulatory domains direct *Abd*-*B* expression in PS10, PS11, PS12, and PS13, respectively (Fig. 1a) [28, 29].

Boundary elements like *Fab*-*7* must function during the two phases of BX-C regulation, initiation, and maintenance [30–32]. During the initiation phase the activity state, *on* or *off*, of the regulatory domains is set by the interactions of gap and pair-rule gene proteins with parasegment-specific initiators. For example, in PS11, the *iab*-*6* initiator turns *iab*-*6 on*, while the initiator in the adjacent domain, *iab*-*7*, sets *iab*-*7* in the *off* or silenced state. In PS12 cells, which have a different combination of gap and pair-rule gene products, the *iab*-*7* initiator activates the *iab*-*7* domain. During this phase, BX-C boundaries block crosstalk between the parasegment initiators in adjacent domains. Once the products of the gap and pair-rule genes disappear, BX-C regulation switches to the maintenance phase in which the activity state established during the initiation phase, either *on* or *off*, is maintained through the action of Trithorax (Trx) and Polycomb-group (PcG) proteins, respectively [33, 34]. These proteins interact with special elements in each domain: Trithorax response elements (TREs) and Polycomb response elements (PREs) [35, 36]. During this phase, BX-C boundaries prevent the spread of chromatin-dependent activation or repression.

The *Fab*-*7* boundary spans a DNA sequence of ~ 1.2 kb and contains three nuclease hypersensitive regions: HS*, HS1, and HS2 [37, 38]. Located next to HS2 is a fourth nuclease hypersensitive region, HS3, which corresponds to the *iab*-*7* PRE [39, 40]. ChIP-Seq experiments indicate that Insv interacts with four sequences (P1-P4) within the *Fab*-*7* boundary [21]. Two of these, P1 and P3, correspond to a palindrome, CCAATTGG, that is found in many Insv ChIPs, and are located in nuclease hypersensitive sites HS* and HS1, respectively (Fig. 1a). P2 is located on the proximal side of HS1 and is unusual in that Insv binding requires P2 probes of > 60 bp in length. While the relevant sequence motifs in these large P2 probes have not been fully elucidated, we found that Insv binding requires a CCAATAAG motif located at the distal edge of the P2 probes [41]. This CCAATAAG motif corresponds to an Elba recognition sequence. P4 maps to nuclease hypersensitive site HS2; however, the Insv recognition sequence in HS2 has not yet been identified [41].

Crystallographic studies have shown that the Insv BEN domain binds to the palindrome CCAATTGG as a dimer with each BEN domain making a similar set of contacts with the palindrome sequence [20]. For this reason, it is not easy to imagine how an Insv BEN domain dimer would be able to interact specifically with the CCAATAAG motif at one end of the P2 probes and some other sequence(s) 60 or more bp apart. A hint as to a possible mechanism came from gel filtration experiments of nuclear extracts. We found that Insv DNA-binding activity fractionates as a very large ~ 420-kDa complex [41]. As Insv monomers are 42 kDa, a dimeric Insv complex should have an apparent molecular weight of only ~ 80 kDa. The 420-kDa complex we detected could contain as many as 10 Insv monomers, or 5 dimeric BEN DNA-binding domains. A multimeric complex of this sort could be relevant to boundary function as it should be able to bind simultaneously to several recognition sequences and potentially link distant elements. It could also explain how Insv binds to the extended P2 probes.

In the studies reported here, we have used a combination of genetic, molecular, and biochemical approaches to explore the biological and biochemical properties of the Insv protein. We show that like the Elba factor, Insv assembles into a multimeric complex in vitro. There are two domains in the Insv protein that mediate multimerization. One of these is a coiled-coil domain (CC) near the N-terminus, while the other is a ~ 45-amino acid domain in the C-terminal half of the Insv protein, just upstream of the Insv BEN domain. The N-terminal domain also contains a 15-amino acid sequence that mediates interactions with CP190. We used a functionally compromised *Fab*-*7* mutant background to explore *insv* boundary function in vivo. In the sensitized genetic background, an *insv* mutation completely abrogates all residual boundary function. As expected, we found that a transgene carrying the *insv* genomic sequence fully rescued the effects of the *insv* mutation, restoring the functioning of the sensitized *Fab*-*7* boundary to that observed in an otherwise wild-type background. Unexpectedly, expressing a wild-type *insv* cDNA with a constitutively active *ubiquitin* (*ubi*) promoter not only rescued the *insv* mutation, but also fully rescued the defective *Fab*-*7* boundary. We used the *ubi*-driven construct to test the functioning of Insv proteins lacking either the CP190 interaction domain, or the N-terminal multimerization domain. A transgene expressing an Insv protein lacking the CP190 interaction domain, *Insv* ^*d135-150*^, also rescued the boundary defects in *insv* mutant flies, but rescue was incomplete. Finally, a transgene expressing an Insv protein, *insv*^*d50-79*^, lacking part of the N-terminal coiled-coil multimerization domain, was compromised for boundary function.

## Results

### Multimerization and DNA-binding activity of Insv

#### Insv protein sequences required for DNA binding

DNA binding by the Elba BEN domain proteins, Elba1 and Elba2, requires a third protein, Elba3, to link them together [25]. In contrast, we found that Insv expressed by in vitro translation in rabbit reticulocyte lysates is able to bind CCAATTGG palindrome without the addition of any co-factors [41]. This finding indicates that Insv must be able to assemble into a dimer that can bind DNA on its own. To confirm this inference and to biochemically map functional domains in the protein, we expressed full-length and truncated versions of the Insv protein in bacteria (Fig. 1b). The bacterially expressed proteins had N-terminal GST (26 kDa), His-Sumo (HS, 11 kDa), or His-Thioredoxin (HT, 13 kDa) tags.

EMSAs of a 32-bp probe containing the P3 CCAATTGG palindrome with different Insv protein variants are shown in Fig. 1c. All of the fusion proteins in panel c have the N-terminal GST tag, while the fusions in panel d have either the GST or HS tag. As expected, full-length Insv (F-Insv) shifts the P3 probe, and as observed in nuclear extracts, it gives a closely spaced doublet (red and black arrows). The GST versions of the N-terminally truncated proteins, C-BEN (Ce-BEN) and BEN, also give shifts, while GST fusions of the N-terminal 213 amino acids (not shown) and the C domain (C) do not. Since in vitro translated full-length Insv can bind to the P3 palindrome, we expected that the bacterially expressed full-length Insv would also be able to dimerize on its own. However, the N-terminal GST tag is known to form dimers, and it could mediate dimerization, and consequently, DNA binding of the truncated proteins in panel b, C-BEN, Ce-BEN, and BEN. To test this possibility, we compared the DNA-binding activity of the GST fusion proteins with the corresponding HS-tagged variants. Unlike GST, the HS tag does not mediate dimerization. Panel d shows that both C-BEN fusion proteins shift the P3 probe. In contrast, only the GST:BEN fusion protein generates a shift. This finding indicates that the BEN domain by itself is unable to bind DNA in solution because it cannot form a stable dimer. However, the addition of the forty-four-amino acid C domain (C-BEN) is sufficient to substitute for the dimerization activity of GST. It is interesting to note that like the full-length protein, two shifts are generated by the HS:C-BEN. The more rapidly migrating shift (red arrow) should correspond to C-BEN dimer, in which case the slower migrating shift (black arrow) would presumably be a tetramer or higher-order multimer.

#### C-BEN and F-Insv assemble into multimeric complexes

**Fig. 2:**
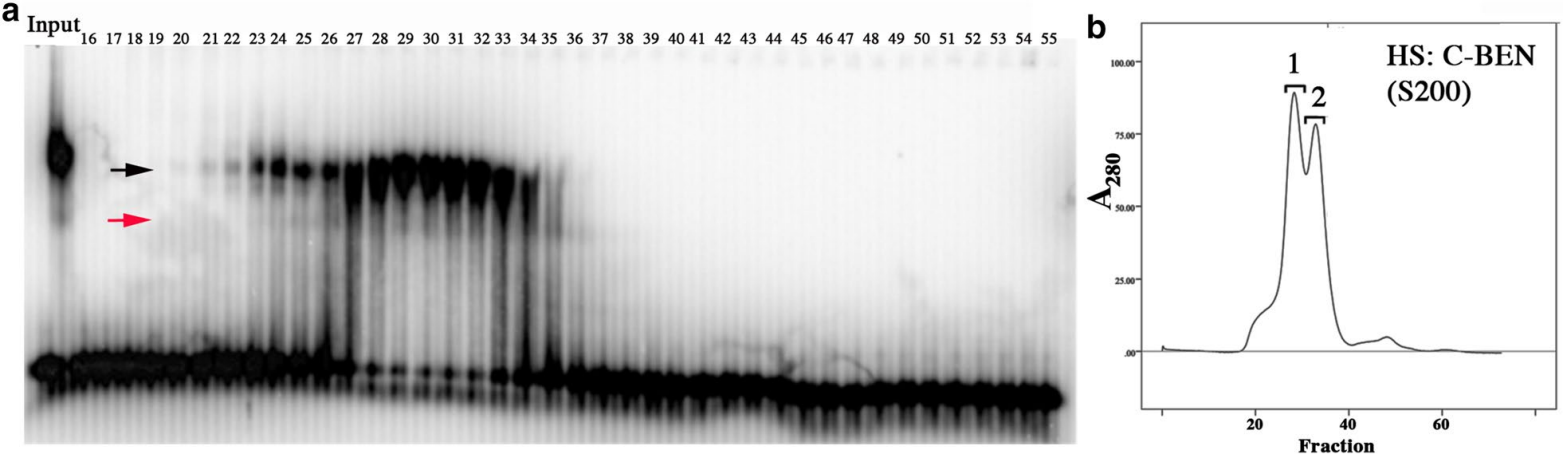
Superose S200 fractionation of HS:C-BEN. **a** His-Sumo C-BEN (HS:C-BEN) was fractionated on a Superpose S200 column, and the column fractions were then assayed for DNA-binding activity by EMSA using the 32-bp P3 probe. Arrows indicate the two shifts generated by HS:C-BEN. **b** *A*_280_ profile of proteins eluted from the Superpose S200 column. Peak 1 has an estimated molecular weight of 190–120 kDa, while the estimated molecular weight of the peak 2 is ~ 60–80 kDa

The findings in the previous section indicate that DNA binding (in solution) by the Insv BEN domain requires a domain that can mediate dimerization. Moreover, since the C-BEN proteins are able to bind DNA in the absence of a chimeric dimerization domain, it would appear that amino acid sequences between 209 and 253 are responsible, at least in part, for dimerization. To test this idea further, we size-fractionated HS:C-BEN using a Superdex 200 gel filtration resin. The A_280_ profile in Fig. 2b shows that there are two peaks of HS:C-BEN. The estimated size of the first peak (1) is ~ 190–120 kDa, while the estimated size of the second peak (2) is ~ 60–80 kDa. Since HS:C-BEN is 32 kDa, the first peak would be expected to correspond to a hexamer and/or tetramer, while the second peak is expected to correspond to a dimer. The DNA-binding profile of the HS:C-BEN protein spans both of these peaks (Fig. 2b).

**Fig. 3:**
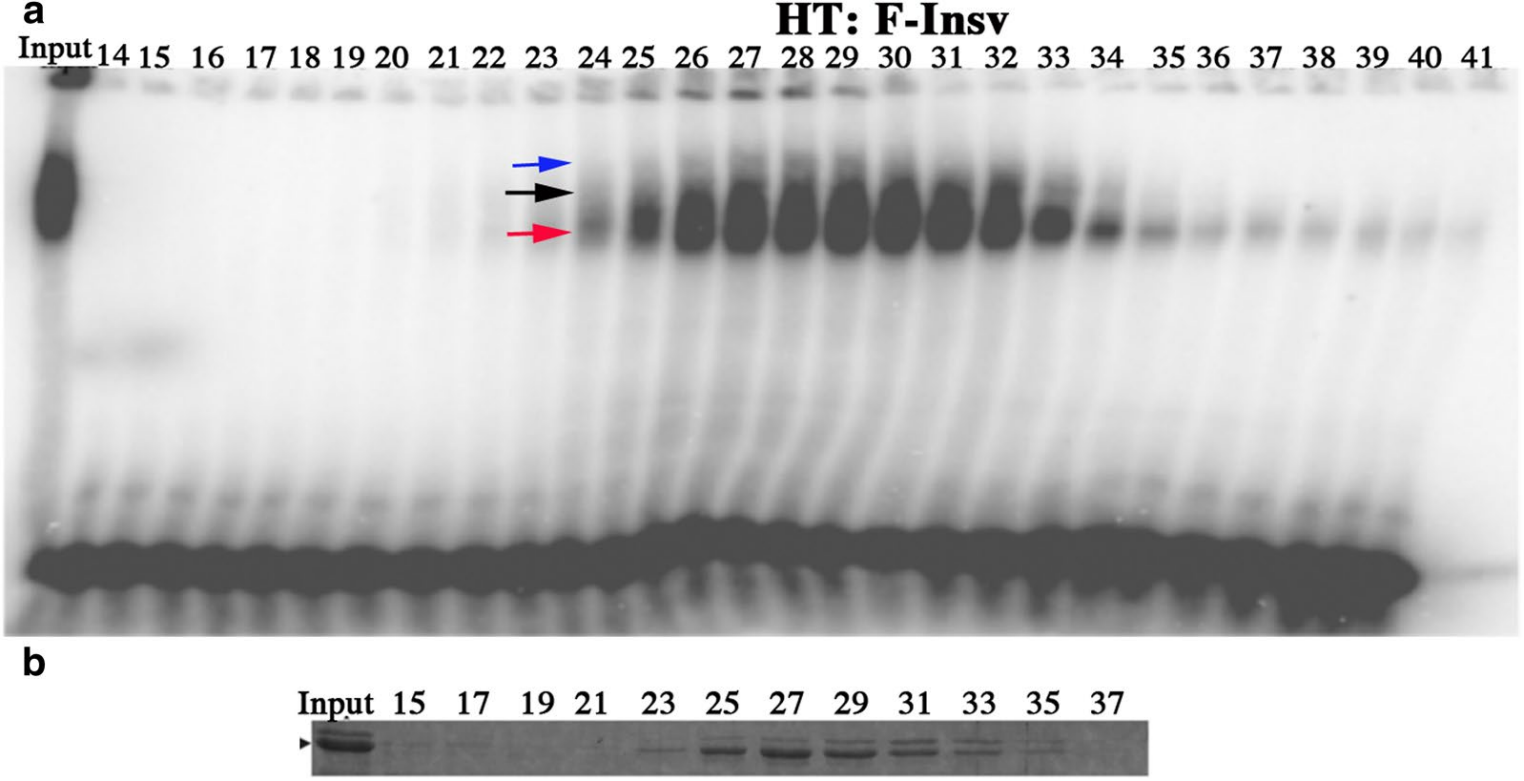
Superose 6 10/300 fractionation of HT:F-insv. His-Thioredoxin F-Insv (HT:F-Insv) was fractionated on a Superose 6 10/300 column, and the column fractions were then assayed for DNA-binding activity by EMSA using the 32-bp P3 probe. **a** DNA-binding activity elutes in a broad peak with an estimated molecular weight ranging from ~ 320 kDa to 120 kDa. Arrows indicate the different shifts that are generated by the HT:F-Insv protein. **b** Fractions from the gel filtration column, as indicated, were analyzed by SDS-PAGE, and the gel was stained with Coomassie to visualize HT:F-Insv

For gel filtration of the larger full-length Insv protein, F-Insv, we used a Superose 6 10/300 size exclusion column. We found that the His-Sumo F-Insv (HS:F-Insv) protein tended to form large aggregates that had minimal DNA-binding activity. However, the peak fractions for DNA-binding activity (but not protein) eluted in broad peak range from ~ 420 to 120 kDa (Additional file 1: Fig. 1). Aggregates were not observed for the 65-kDa His-Thioredoxin F-Insv (HT:F-Insv) fusion. In this case, both the protein and DNA-binding activities were closely matched and eluted as a broad peak between 340 and 120 kDa (Fig. 3). Thus, bacterially expressed F-Insv appears to assemble into a mixture of dimers and tetramers. Additionally, there is a trail of monomers in the later eluting fractions. While these findings indicate that multimerization is an intrinsic property of Insv, it should be noted that the complexes assembled by the full-length bacterially expressed protein are smaller than those detected in nuclear extracts (~ 420 kDa), and apparently much less stable.

#### DNA-binding activities of C-BEN and F-Insv

The BEN domain interacts with DNA as a dimer. Thus, the formation of multimeric complexes containing either four or six Insv proteins could generate either two or three BEN domain dimers. In principle, each of these BEN domain dimers could interact with a distinct DNA sequence. This could explain (at least in part) why the Insv protein in nuclear extracts is able to bind to the large (> 60-bp) P2 probes, even though they lack the preferred Insv palindromic sequence (CCAATTGG). The Insv complex would be anchored at one end by a BEN dimer bound to the Elba recognition sequence, and at the other end by one or more BEN dimers bound to sequences up to 60 or more bp distant. If this is correct, then the bacterially expressed protein should on its own be able to bind P2 probe. Since the truncated C-BEN protein forms not only dimers, but also tetramers and/or hexamers, we examined its binding activity as well.

**Fig. 4:**
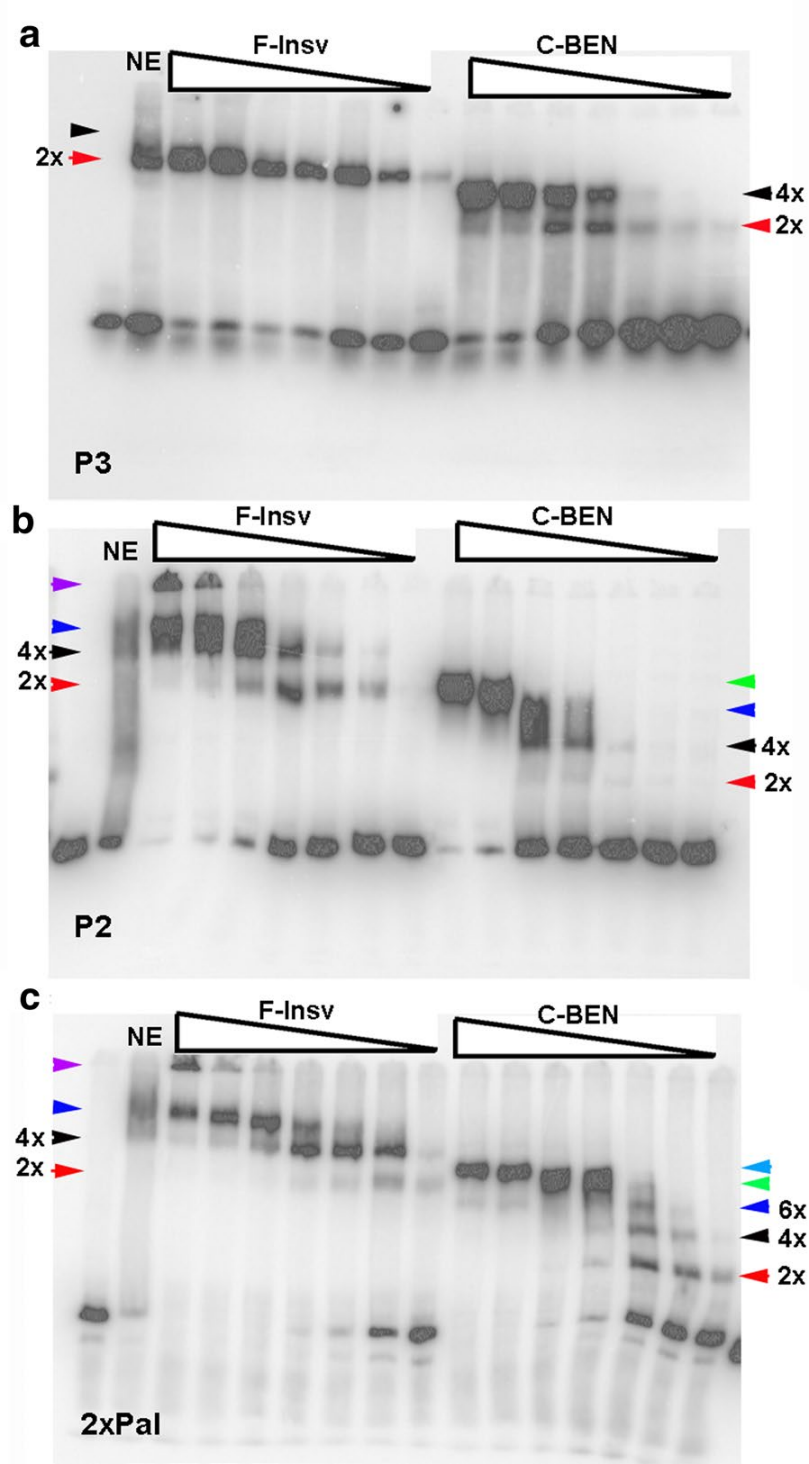
HT:F-Insv and HS:C-BEN generate complex patterns of shifts with different substrates. **a** HT:F-Insv and HS:C-BEN shifts of probe P3. **b** probe P2 (see diagram in Fig. 1). **c** probe 2xPal (see diagram in Fig. 1). Predicted multimers are indicated by arrowheads (see also text). Amounts of protein added (left to right) to the reaction mix were estimated based on the relative intensity of the Coomassie-stained protein bands in SDS-PAGE gels: 0.5 μM, 0.25 μM, 0.05 μM, 0.025 μM, 0.005 μM, 0.0025 μM, and 0.0005 μM

We first examined the DNA-binding activities of HT:F-Insv and HS:C-BEN using the 32-bp P3 probe (Fig. 1a). The HT:F-Insv:P3 combination gives a heavily labeled shift (red arrowhead, Fig. 4a). While one or two additional, more slowly migrating shifts are typically observed for HT:F-Insv (cf. Fig. 3), these secondary shifts are only weakly labeled in this particular experiment. Based on gel filtration (see above) and cross-linking experiments (see below), the heavily labeled band is likely to be generated by an HT:F-Insv dimer. In the case of the truncated protein HS:C-BEN, there are two P3 shifts. The more rapidly migrating shift (red arrowhead) is expected to correspond to the HS:C-BEN dimer, while the more slowly migrating shift (black arrowhead) should be the tetramer/hexamer. As would be expected from the studies discussed above, the ratio of these two shifts is concentration dependent: the slower shift predominates at higher concentrations.

More complicated patterns are observed for the 117-bp P2 probe (Fig. 1a). For HT:Insv, two shifts are observed at lower protein concentrations (Fig. 4b). As the input protein appears to be a mixture of dimers and tetramers, the more rapidly migrating shift (red arrowhead) is most likely an HT:F-Insv dimer, while the more slowly migrating shift (black arrowhead) would be generated by an HT:F-Insv tetramer. At higher protein concentrations, the most rapidly migrating shift disappears (red arrowhead) and is replaced by a more slowly migrating shift (blue arrowhead) which would correspond to a higher-order multimer (Fig. 4b). The black and blue shifts resemble the pair of shifts seen for the P2 probe in nuclear extracts (NE: Fig. 4b) [41]. In addition, at high protein concentrations, a significant fraction of the probe fails to enter the gel (purple arrowhead). As the input protein is largely a mixture of dimers and tetramers, it would appear that the formation of these larger complexes may be stimulated by DNA binding. The truncated HS:C-BEN also gives multiple shifts with the P2 probe (Fig. 4b). The most rapidly migrating shift (2x, red arrowhead) is present in very low yield and is expected to correspond to an HS:C-BEN dimer, while the second much more heavily labeled shift (4x, black arrowhead) corresponds to the tetramer/hexamer. Like HT:F-Insv, HS:C-BEN also generates additional shifts (blue and green arrowheads) that presumably correspond to higher-order multimers (e.g., 3x and 4x dimers).

We also tested a 122-bp probe, 2xPal (see Fig. 1), which has two CCAATTGG palindromes. As was observed with the P2 probe, HT:F-Insv generates at least three distinct shifts with the 2xPal probe (Fig. 4c). The most rapidly migrating shift is expected to correspond to a single dimer (red arrowhead), while the other more slowly migrating shifts (black and blue arrowheads, plus the complexes trapped at the top of the gel) are expected to correspond to different combinations of dimers and tetramers bound to the two CCAATTGG palindromes in the 2xPal probe. HS:C-BEN also generates multiple shifts. Unlike P2, HS:C-BEN dimers (red arrowhead) are readily detected. Moreover, dimer (red arrowhead) and various multimeric combinations (e.g., dimer + dimer, dimer + tetramer, tetramer + tetramer: black, dark blue, green, light blue arrowheads) appear at much lower protein concentrations. This pattern is expected since unlike P2, the 2xPal probe has two consensus Insv recognition sequences.

##### Identification of Insv domains involved in self-association (multimerization)

**Fig. 5:**
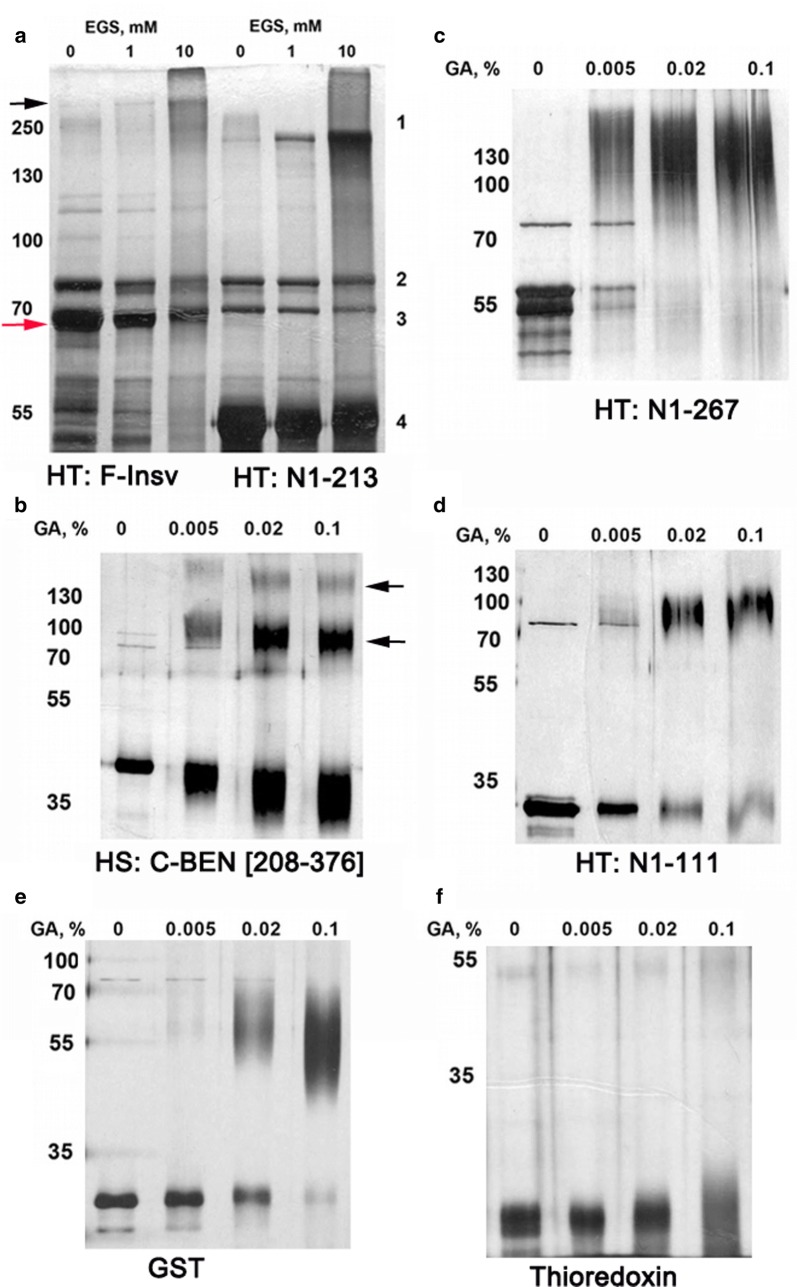
Detection of multimeric Insv complexes by chemical cross-linking. **a** Cross-linking of Thioredoxin-fused full-length Insv and 1-213-aa fragment with EGS. **b**–**d** Cross-linking of Thioredoxin-fused Insv protein fragments, as indicated, with glutaraldehyde. Protein sample concentrations in the cross-linking reaction mix ranged from 0.3 μg/mL to 1.1 μg/mL. **e**, **f** Cross-linking of controls, GST, and Thioredoxin. GST generates a dimer, while Thioredoxin remains monomeric

We used chemical cross-linking to provide additional evidence that Insv assembles into multimeric complexes and to further localize amino acid sequences contributing to protein–protein interactions. We first cross-linked the full-length HT:F-Insv with increasing concentrations of a bifunctional cross-linker, EGS (ethylene glycol bis(succinimidyl succinate)). The migration of the denatured 55-kDa HT:F-Insv protein in SDS-PAGE is just slightly faster than the 70-kDa marker (Fig. 5a, red arrow). The addition of increasing amounts of EGS generates a band of nearly 300 kDa (black arrow). This is close to that expected for a tetrameric complex and would be in agreement with gel filtration experiments.

Gel filtration and DNA-binding experiments with N-terminal truncations show that C-BEN, which lacks amino acids 1-209, is able to bind DNA because it can multimerize, forming a mixture of dimers and tetramers. This conclusion is supported by glutaraldehyde cross-linking (Fig. 5b). Denatured HS:C-BEN (~ 32 kDa) has an apparent molecular weight of about 42 kDa. In the presence of cross-linker, two additional bands, one of about 90 kDa (bottom arrow) and another of about 160 kDa (top arrow) are observed. The former would correspond to a dimer, and the latter to a tetramer.

Taken together with our gel filtration and EMSA experiments, these findings indicate that the C domain can direct the assembly of dimeric as well as multimeric complexes. However, we suspected that there are additional protein–protein interaction domains in Insv. To test this possibility, we generated two C-terminal truncations, HT:N1-267 and HT:N1-213. HT:N1-267 includes the entire C domain plus the first 14 amino acids of the BEN domain, while all but the first four amino acids of the C domain are deleted in HT:N1-213. Though they differ in length, both HT:N1-267 and HT:N1-213 migrate at an apparent molecular weight of about 55 kDa on SDS-PAGE gels (Fig. 5a, c, respectively). After cross-linking, each gives an additional band of ~ 200 kDa, as would be expected for a tetramer. Consistent with these results, the estimated size of HT:N1-213 in gel filtration experiments is about 190 kDa. These findings indicate that sequences in the N-terminal domain can also mediate multimer formation.

Protein structure prediction programs model a coiled-coil domain in the region spanning amino acids 51-104 [42]. The precise endpoints of the domain vary depending upon the prediction program. Moreover, this coiled-coil domain is also predicted to self-associate as a tetramer [43]. To test the possible involvement of this predicted coiled-coil domain in Insv multimerization, we cross-linked a C-terminal truncation, HT:N1-111, which includes the predicted coiled-coil domain. Figure 5d shows that cross-linking of the ~ 25-kDa HT:N1-111 protein generates a product that has a mobility consistent with that expected of a tetramer.

**Fig. 6:**
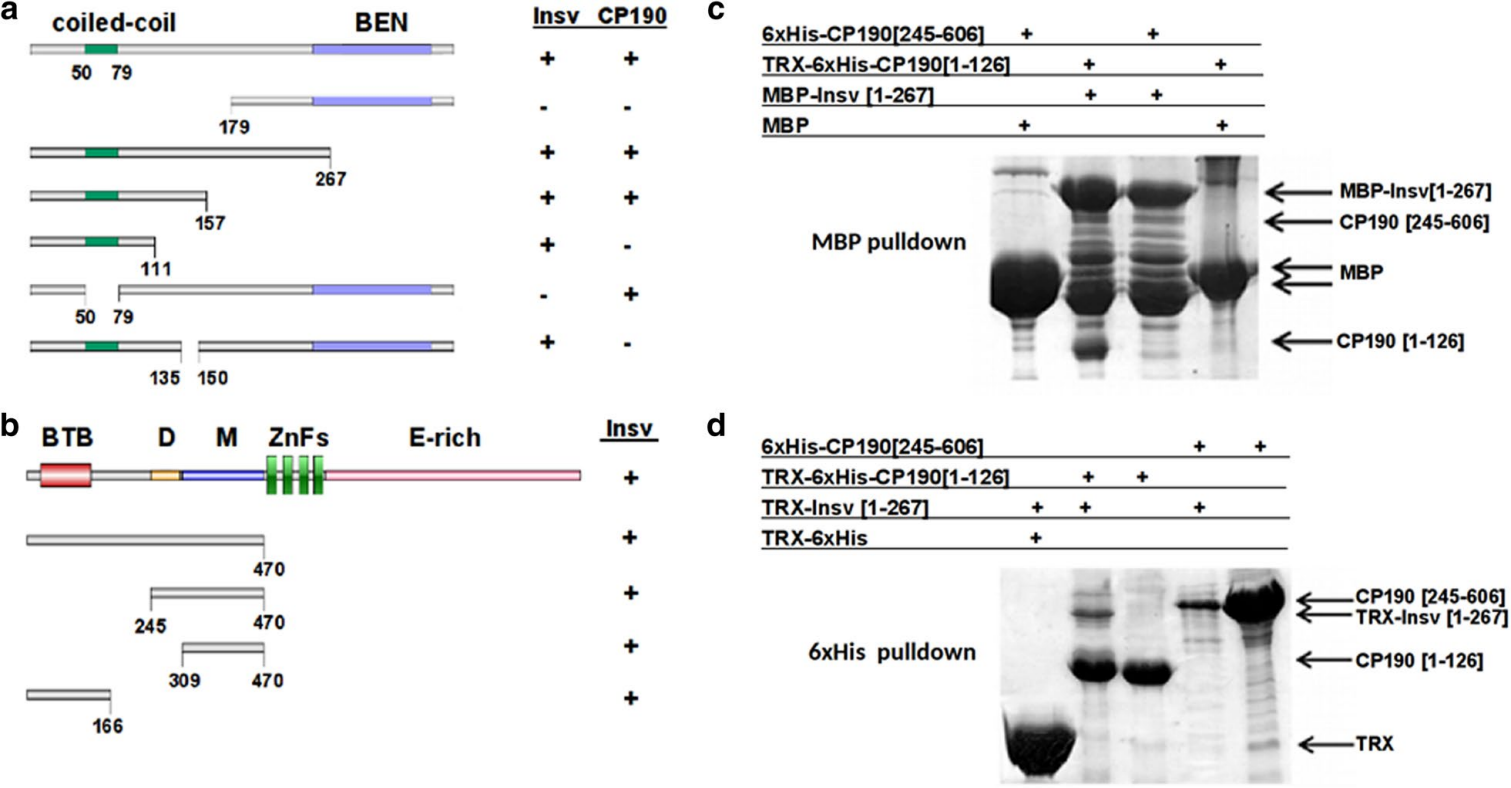
Insv–Insv and Insv–CP190 interactions. **a** Summary of two-hybrid experiments with full-length and truncated Insv proteins with either full-length Insv or full-length CP190. Positive interactions are indicated by (+) and negative by (−). **b** Summary of two-hybrid experiments with full-length or truncated CP190 proteins and full-length Insv. **c**, **d** Reciprocal pull-down experiments with tagged truncated Insv and CP190 proteins, as indicated

To complement these biochemical experiments, we used the yeast two-hybrid system to identify interacting domains in the Insv protein. For this purpose, we fused full-length Insv to the Gal4 DNA-binding domain (Gal4DB) and different sequences from Insv to the Gal4 activation domain (Gal4AD). The results of this analysis are summarized in Fig. 6a. While the full-length Insv and proteins containing the N-terminal half of the Insv protein activate transcription, the Insv derivative containing just the C-terminal C-BEN sub-fragment did not. Since bacterially expressed C-BEN forms dimers and tetramers, it is not entirely clear why this protein does not interact with the full-length Insv in the yeast two-hybrid assay. One possibility is that the interaction is too weak to generate stable Gal4DB-Gal4AD complexes. Alternatively, it is possible that the configuration of the Gal4DB:Insv–Gal4AD:C-BEN complex might not be conducive for transcriptional activation. We also found that an Insv deletion mutant, Insv d50-79, which lacks part of the predicted coiled-coil domain is unable to interact with the full-length Insv.

##### Insv directly interacts with CP190

Experiments by Dai et al. [21] showed that Insv interacts with the boundary protein CP190 in nuclear extracts. We used the yeast two-hybrid system to confirm that the interaction is direct and to map the sequences in Insv and CP190 that mediate interactions. The experiments summarized in Fig. 6a show that a 15-amino acid sequence in the N-terminal half of Insv (135-150) is required for Insv:CP190 interactions. In the case of CP190 (Fig. 6b), we found that two regions in the N-terminal half of the CP190 protein, aa 1-166 and aa 309-470, can independently interact with full-length Insv. The former contains the CP190 BTB domain, while the latter has the M domain [42–44].

To provide further evidence for direct interactions, we used pull-down experiments with bacterially expressed proteins. In Fig. 6c, maltose-binding protein (MBP) or a maltose-binding protein fused to Insv 1-267 (MBP-Insv[1-267]) were used to pull down fusion proteins containing different regions from the N-terminal half of CP190. The MBP-Insv[1-267] fusion protein (but not the MBP protein) pulled down a His-tagged fusion protein containing the CP190 N-terminal BTB domain (TRX-6xHis-CP190 [1-126]). In the reciprocal experiment (Fig. 6d), TRX-6xHis-CP190 [1-126] pulled down the TRX-Insv [1-267] fusion protein. In contrast, we were unable to detect interactions between TRX-Insv [1-267] and a His-tagged CP190 protein spanning the M domain (TRX-6xHis-CP190 [245-606]).

##### Functional analysis of Insv proteins lacking the coiled-coil or CP190 interaction domains

**Fig. 7:**
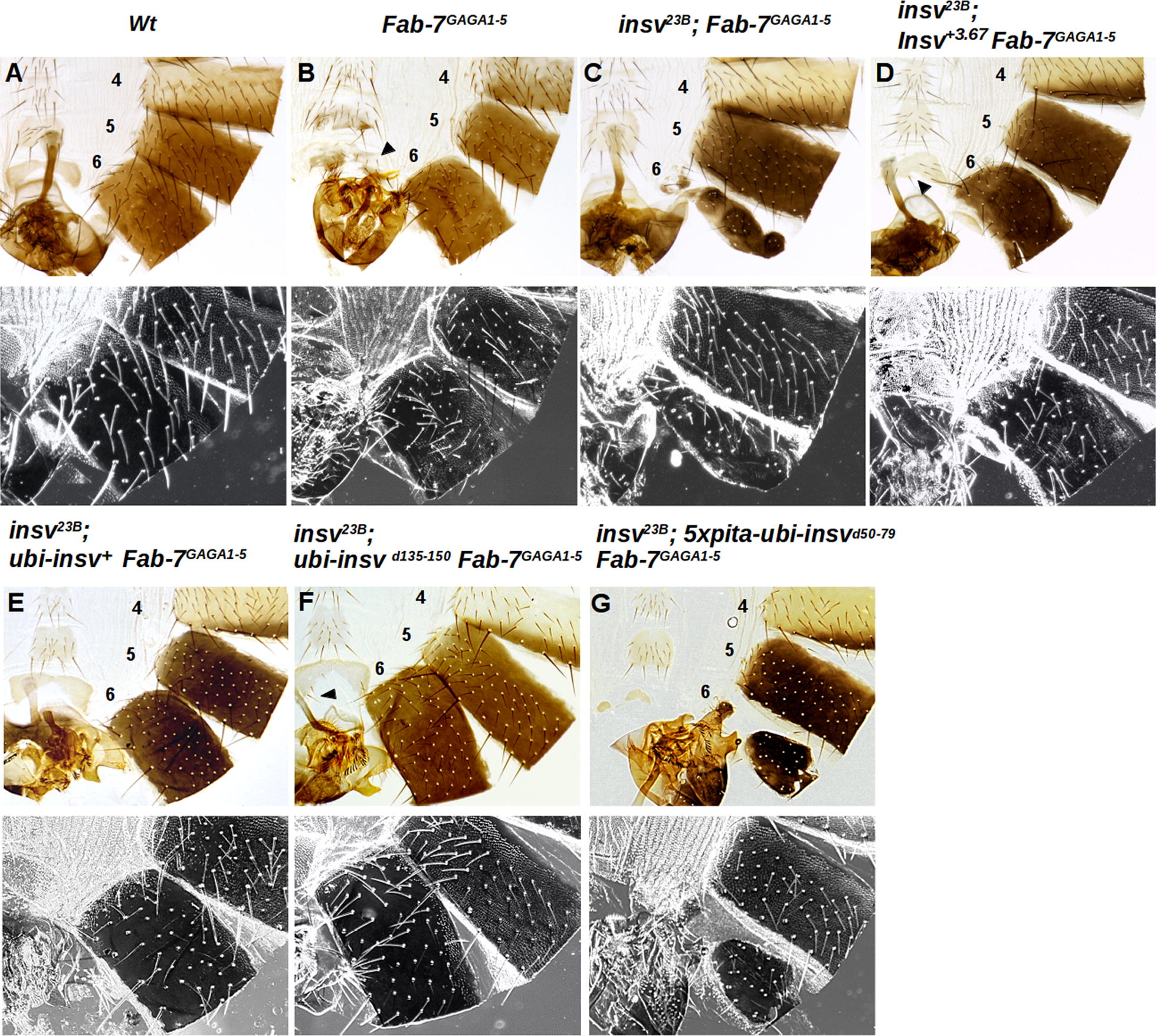
In vivo architectural functions of wild-type and mutant Insv proteins. **A** Adult abdominal cuticle preparations of a wild-type male. The fifth and sixth tergites are pigmented, and the A6 sternite is recognizable by the absence of bristles and a banana shape. Trichomes are visible in the dark field (black and white lower panels) and cover the entire surface of the A5 tergite, while for the A6 tergite the trichomes are found in thin stripes along the anterior and ventral edges of the A6 tergite. **B** Homozygous males with a sensitized genetic background of Fab-7: *Fab*-*7*^*GAGA1-5*^ (for description see the text). **C** When the *insv* null allele, *insv*^*23B*^, is combined with *Fab*-*7*^*GAGA1-5*^, this disrupts the boundary function of the *Fab*-*7*^*GAGA1-5*^ replacement and A6 is misspecified. Males have a mixed GOF and LOF phenotype. A6 is partially transformed into A7 (GOF transformation), but at the same time, the residual A6 cuticle has morphological features characteristic of A5 (trichomes visible in the dark field, LOF transformation). **D** Rescue of the mutant *insv*^*23B*^; *Fab*-*7*^*GAGA1-5*^ phenotype with a genomic rescue construct *insv*^+*3.67*^. The genomic Insv fragment restores the boundary function of *Fab*-*7*^*GAGA1-5*^. The A6 tergite is only marginally smaller than wild type, and the trichomes are usually limited to small patches. **E**–**G** Show the abdominal cuticles of males carrying the *ubi* rescue constructs in an *insv*^*23B*^*; Fab*-*7*^*GAGA1-5*^ background. **E** Wild-type *ubi*-*insv*^+*3.67*^ rescues *all Fab*-*7*^*GAGA1-5*^ boundary defects. Males have tergites and sternites that are indistinguishable from the wild type. **F** Rescue obtained with *ubi*-*insv*^*d135-150*^ construct. Although *insv*^*23B*^*; ubi*-*insv*^*d135-150*^ *Fab*-*7*^*GAGA1-5*^ homozygotes look almost wild type, A6 sternites typically have two or more bristles, indicative of a weak A6 to A5 transformation. **G** Rescue by the coiled-coil domain deletion transgene *5xpita*-*ubi*-*insv* ^*d50-79*^ is incomplete. The A6 tergites in *insv*^*23B*^*; 5xpita*-*ubi*-*insv* ^*d50-79*^ *Fab*-*7*^*GAGA1-5*^ males are reduced in size and are irregularly shaped, as expected for a strong GOF transformation. The tergites also have small patches of trichome hairs in regions where they are absent in wild type, indicating that it is also a LOF transformation. Sternites are also much smaller than in *Fab*-*7*^*GAGA1-5*^ and unlike *Fab*-*7*^*GAGA1-5*^ are misshapen or absent altogether. The numbers 4, 5, and 6 indicate A4, A5, and A6 abdominal segments, respectively

Previous studies have implicated Insv in *Fab*-*7* boundary activity [21, 41]. *Fab*-*7* is composed of multiple functionally redundant elements, and consequently, the *insv* null mutation, *insv*^*23B*^, has no obvious effects on *Fab*-*7* function. For this reason, we took advantage of a sensitized background in which *Fab*-*7* was replaced by a mutant boundary, *Fab*-*7*^*GAGA1-5*^. In this replacement, mutations were introduced in five of the six GAGA factor (GAF)-binding sites (GAGA1-5) in the large HS1 hypersensitive region. *Fab*-*7*^*GAGA1-5*^ retains boundary function, and about 15% of the replacement male flies resemble wild type. However, in the remaining animals there are weak and variable alterations in segment morphology. In most male *Fab*-*7*^*GAGA1-5*^ flies, the A6 tergite is marginally reduced in size as would be expected for a weak gain-of-function (GOF) transformation (Fig. 7B). At the same time, there are small patches of trichomes in regions of the A6 tergite that should be devoid of them. This is characteristic of a loss-of-function (LOF) transformation of A6 (PS11) into A5 (PS10). The sternites also show a mixture of weak GOF and LOF. When the *insv*^*23B*^ mutation is introduced into the *Fab*-*7*^*GAGA1-5*^ background, boundary activity is completely abrogated and the segment morphology resembles that observed in a deletion that removes the three *Fab*-*7* hypersensitive sites: HS*, HS1, and HS2 [46].

To determine whether the two protein–protein interaction domains in the N-terminal half of the Insv protein are functionally important in vivo, we generated rescue constructs in which either the wild-type protein or proteins lacking these domains are expressed under the control of the Ubiquitin (*ubi*) promoter. These rescue constructs were inserted at the same chromosomal location using the phiC31 integration system. We first tested their ability to rescue the boundary defects of the *insv*^*23B*^; *Fab*-*7*^*GAGA1-5*^ combination.

The first and unexpected finding was that the rescuing activity of *ubi*-*insv*^+^(Fig. 7E) was different from what we have observed previously for a genomic rescue construct *insv*^+*3.67*^ (Fig. 7D; [41]). The genomic transgene fully rescued the effects of the *insv* mutation on the *Fab*-*7*^*GAGA1-5*^ boundary, and the spectrum and frequency of phenotypes observed for *insv*^*23B*^; *insv*^+*3.67*^ *Fab*-*7*^*GAGA1-5*^ flies were the same as that for the *Fab*-*7*^*GAGA1-5*^ mutant in a wild-type *insv*^+^*/insv*^+^ background (Fig. 7B). By contrast, the *ubi*-*insv*^+^ construct fully rescued *all* *Fab*-*7*^*GAGA1-5*^ boundary defects, and *insv*^*23B*^; *ubi*-*insv*^+^ *Fab*-*7*^*GAGA1-5*^ flies resembled *wild type* not *Fab*-*7*^*GAGA1-5*^ flies (Fig. 7E). One explanation for this discrepancy is that the *ubi* rescue construct is more broadly expressed than either the endogenous *insv* gene or the genomic rescue construct. The endogenous *insv* is active throughout the embryo at the blastoderm stage; however, during germband retraction, expression becomes progressively restricted to a subset of cells in the CNS and PNS [21]. This seems to be true for the genomic rescue construct as well (see Additional file 1: Fig. S2). In contrast, at this stage in development, the Insv protein expressed by *ubi*-*insv*^+^ rescue transgene is detected in cells outside of the CNS and PNS. While we have not made a careful comparison of the expression patterns later in development, we have found that the *ubi* rescue transgenes express Insv in salivary glands (see below), while the protein encoded by the endogenous *insv* gene is not found in this tissue.

The *ubi*-*insv* ^*d135-150*^ transgene, which expresses an Insv protein that has a deletion of the Insv:CP190 interaction domain, also rescues the boundary defects of *insv*^*23B*^; *Fab*-*7*^*GAGA1-5*^ flies. A typical example of an *insv*^*23B*^*; ubi*-*insv*^*d135-150*^ *Fab*-*7*^*GAGA1-5*^ male is shown in Fig. 7F. As is observed for the *ubi*-*insv*^+^ transgene, *ubi*-*insv*^*d135-150*^ males have tergites that are indistinguishable from wild-type males. Moreover, unlike *insv*^*23B*^; *Fab*-*7*^*GAGA1-5*^ males rescued by the genomic transgene *insv*^+*3.67*^, the A6 trichome pattern in *insv*^*23B*^*; ubi*-*insv*^*d135-150*^ *Fab*-*7*^*GAGA1-5*^ males is wild type. However, the rescuing activity of the *ubi*-*insv*^*d135-150*^ transgene is not equivalent to the *ubi*-*insv*^+^ transgene as the A6 sternites typically have two or more bristles, indicative of a weak PS11 → PS10 transformation. Given the fact that the *ubi*-*insv*^+^ transgene is more effective than two copies of the endogenous *insv* gene (or the *insv*^+*3.67*^ transgene), it seems likely that the functional capabilities of the Insv d135-150 protein are slightly impaired.

We found that the coiled-coil deletion in *ubi*-*insv*^*d50-79*^ transgene, at most, only marginally rescues the *insv*-dependent defects in boundary function in *insv*^*23B*^; *Fab*-*7*^*GAGA1-5*^ mutant flies; however, Western blots showed that the expression of the mutant protein is reduced compared to the wild-type Insv expressed under the *ubi* promoter. For this reason, we generated a new *ubi*-*insv*^*d50-79*^ rescue construct that has multimerized binding sites for the Pita boundary protein upstream of the promoter. Although the level of protein expressed by the *5xpita*-*ubi*-*insv*^*d50-79*^ construct is equivalent to that of the *ubi*-*insv*^+^ construct (Additional file 1: Fig. S3), rescue is still minimal. As illustrated in Fig. 7G, the A6 tergites in *5xpita*-*ubi*-*insv*^*d50-79*^ are substantially reduced in size and are irregularly shaped. In fact, they are at most only slightly larger than the typical tergites observed in the *insv*^*23B*^; *Fab*-*7*^*GAGA1-5*^ double mutant (compare Fig. 7C with G). The residual tergites also have small patches of trichome hairs in regions where they are absent in wild type, indicative of a LOF transformation. Morphological abnormalities are also observed in the sternites. Though present somewhat more frequently than in the parental *insv*^*23B*^; *Fab*-*7*^*GAGA1-5*^ double mutant, they are much smaller than in *Fab*-*7*^*GAGA1-5*^ and are always misshapen. Thus, the boundary activity of the N-terminal coiled-coil domain deletion is substantially impaired.

##### Chromosomal association of Insv at some sites is dependent on the CP190 interaction domain

**Fig. 8:**
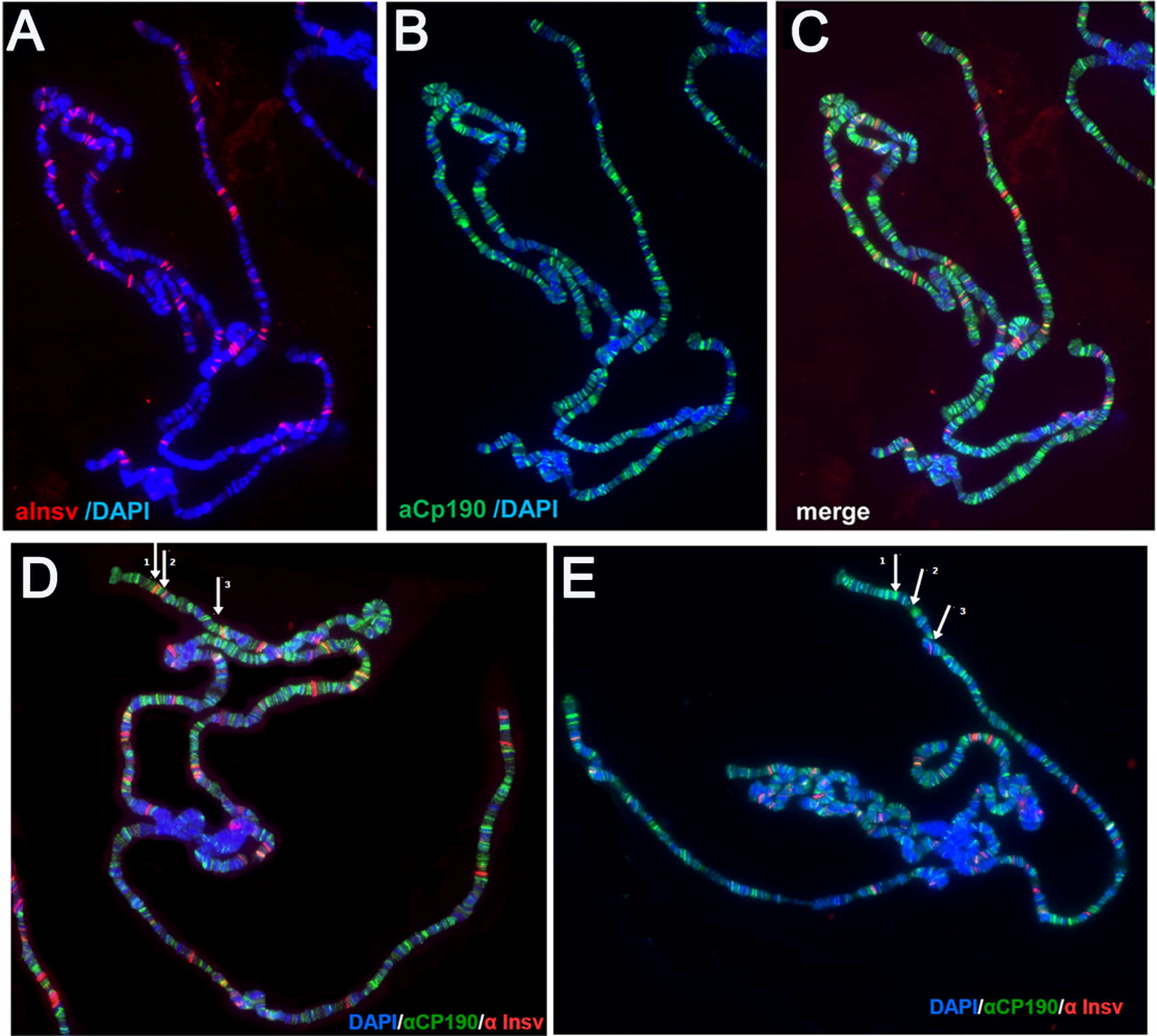
The Insv:CP190 interaction domain is required for the Insv binding to a subset of sites on polytene chromosomes. **A**, **B** Immunostaining of polytene chromosomes from the salivary glands of third-instar *ubi*-*insv*^+^ larvae co-stained with antibodies against Insv (A) and CP190 (B); DAPI staining indicates DNA (in blue). The merged image is shown in **C**. Insv partially localizes to sites of CP190 enrichment. **D** Wild-type Insv localizes to three sites (arrows 1, 2, and 3) near the distal end of chromosome arm 3 L. In contrast to wild type, Insv that has a deletion of the CP190-interacting domain is not observed at sites 1 and 2, while it is still present at site 3 (**E**)

While the findings in the previous section indicate that the CP190 interaction domain is not essential for boundary function in the context of *Fab*-*7*, we found that it is important for recruiting Insv to a subset of sites in polytene chromosomes. Unlike the endogenous *insv* gene, the *ubi* promoter is active in this tissue and Insv protein can be detected in polytene chromosomes. The chromosomal distribution of Insv and CP190 in salivary gland polytene chromosomes is shown in panels A–C of Fig. 8. The number of Insv sites detected in these experiments is considerably fewer than the number of CP190 sites. A comparison of the Insv and CP190 sites (panel C) indicates that a subset of the Insv sites overlaps with CP190 sites. Insv binding to a subset of these CP190-overlapping sites depends upon the Insv:CP190 interaction domain. Panel D shows that wild-type Insv localizes to three sites (1, 2, and 3) near the tip of 3L. For sites 1 and 2, Insv co-localizes with CP190, while it does not co-localize with CP190 at site 3 (panel D). In the *ubi*-*insv*^*d135-150*^ mutant, Insv is not observed at sites 1 and 2, while it is still present at site 3 (panel E).

The finding that Insv association with a subset of sites in polytene chromosomes depends on the CP190 interaction domain prompted us to test the boundary function of the *insv*^*d135-150*^ mutant in a second *Fab*-*7*-sensitized background, *HS1 *+ *HS2*. The reason for choosing this replacement is that though Insv is found associated with HS2 in ChIP experiments, there are no sequences in HS2 that resemble the Insv palindrome and we have not been able to detect Insv binding to HS2 sequences in EMSAs. Thus, it seemed possible that Insv association with HS2 could be mediated at least in part by CP190, in which case the functioning of the *HS1 *+ *HS2* replacement might require the Insv:CP190 interaction domain. However, Additional file 1: Fig. S4 shows that this expectation is incorrect. The *ubi*-*insv*^*d135-15*^ transgene is just about as effective as the *ubi*-*insv*^+^ transgene. This could mean that Insv binding to HS2 does not require CP190. Alternatively, since HS1 contains two sequences (P2 and P3) that are bound by bacterially expressed Insv, they might be sufficient for restoring the boundary activity of Insv ^*d135-15*^ even if the mutant protein cannot bind to HS2. In this context, it is interesting to note that *insv*^*23B*^*; HS1 *+ *HS2* flies carrying the *ubi*-*insv*^+^ rescue transgene resemble *HS1 *+ *HS2* flies, not the wild-type flies as was observed for the *Fab*-*7*^*GAGA1-5*^ mutant. Thus, misexpression of *insv* is unable to “boost” the boundary function of the *HS1 *+ *HS2* replacement.

## Discussion

Boundaries subdivide eukaryotic chromosomes into a series of looped domains (or TADs) by pairing with each other [2, 3, 10]. Though pairing is a characteristic property of these architectural elements in animals from flies to man, the mechanisms responsible for specifying pairing partners in different species are seemingly quite distinct. In mammals, a loop extrusion model is thought to be responsible for determining how boundaries match up [1, 45–50]. In this model, pairing partners are determined by the proximity of convergent sites for CTCF and the action of a molecular machine, the cohesion complex, which brings the convergent CTCF sites together. While this model fits the results of Hi-C experiments in mammalian cells, it does not explain how boundaries pair in flies. For one, CTCF is only one of many DNA-binding proteins that have documented boundary activity in flies. For another, boundary pairing interactions do not depend upon the proximity of convergent CTCF sites. Instead, boundaries that are appropriately “matched” can find and pair with each other over distances spanning hundreds of intervening boundaries (with or without CTCF sites) and TADs [51–55]. Moreover, their ability to pair is completely independent of their relative 5’ → 3’ orientations in the chromosome. Additionally, pairing interactions between fly boundaries occur not only in *cis*, but also in *trans*. *Cis* interactions are heterologous and typically involve boundaries in the same neighborhood. By contrast, *trans* interactions are self-interactions between the same boundaries on each homologue. These self-interactions are responsible for aligning and pairing of homologues in precise register [10, 56, 57].

Although the mechanisms responsible for boundary pairing in flies are not understood in any detail, it is clear that pairing depends upon the identity and properties of the proteins associated with the paired boundaries. Thus far, two different mechanisms have been implicated in pairing interactions. One involves interactions between proteins associated with each boundary. An example of this mechanism would be protein–protein interactions between Zw5 bound to *scs* and BEAF bound to *scs’* [5]. The other mechanism is the deployment of multivalent DNA-binding proteins. The fly dCTCF, which is thought to assemble into tetrameric complexes, would be an example of a bridging factor that could simultaneously bind to single CTCF sites in neighboring boundaries, both in *cis* and in *trans* [58]. Notably, like Zw5 and BEAF, dCTCF also engages in interactions with heterologous proteins [58–60]. A better understanding of how fly boundaries are able to pair selectively with themselves and with their preferred partners requires characterization of the biochemical and biological properties of the identified fly boundary proteins. In the studies reported here we have focused on the BEN domain protein Insv.

The crystal structure of the Insv BEN domain shows that it binds to its 8-bp palindromic recognition sequence as a dimer [20]. The Insv protein is ~ 42 kDa, and thus a dimer is expected to be ~ 80 kDa. However, when we size-fractionated nuclear extracts, we found that Insv DNA-binding activity eluted as a complex of ~ 400 kDa, which would potentially correspond to ~ 10 Insv subunits, or 5 DNA-binding dimers. While this complex could contain other proteins besides Insv, the fact that Insv assembles into such a large complex would be consistent with the idea that it has domains both for self-interactions and for interactions with other proteins. Since these domains could influence how Insv interacts with DNA and contribute to its boundary function, we have attempted to identify them. Experiments with BEN domains tagged with either GST (which dimerizes) or His-Sumo (HS) (which does not dimerize) indicate that the BEN domain is unable to bind to its recognition sequence in solution because it cannot form a stable dimer. Instead, BEN domain dimerization (or multimerization) depends upon sequences in the N-terminal two-thirds of the Insv protein.

We have identified two domains that mediate Insv-self-protein–protein interactions. One is a coiled-coil domain spanning amino acid sequences ~ 51–104 and, depending upon the program, containing 6 or 7 heptad repeats. This coiled-coil domain is predicted to assemble into a tetramer. This predicted tetrameric structure is supported by both chemical cross-linking and gel filtration experiments on N-terminal proteins that contain the putative coiled-coil domain. The second interaction domain, C, is in the C-terminal half of the protein and extends from aa 209 to the beginning of the BEN domain (aa 253). Cross-linking and gel filtration experiments indicate that a derivative protein spanning the C and BEN domains assembles into a mixture of dimers and tetramers.

While these findings indicate that Insv contains two domains which could potentially promote the formation of large multimeric complexes, we did not detect complexes of ~ 420 kDa with bacterially expressed HT-Insv. Instead, the HT-Insv complexes size-fractionated between 120 kDa and 340 kDa, and appeared to be mostly a mixture of dimers and tetramers. Some active complexes close to the size of the Insv complex in nuclear extracts were detected when the bacterially expressed Insv was tagged with HS instead of HT; however, the HS-tagged Insv also tends to form even larger inactive aggregates. There are a number of explanations for this discrepancy. One is that the native Insv complex cannot be fully assembled by the protein folding machinery in bacteria and that when larger multimers are formed they have unstructured regions that promote aggregation. Another possibility is that the large complex in nuclear extracts is stabilized by post-translational modifications that are lacking in the bacterial protein. It is also possible that the endogenous complex includes not only Insv but also other proteins that help to stabilize it. Here, an obvious candidate would be CP190. However, CP190 antibodies had no apparent effect on the Insv shifts generated by nuclear extracts. With the caveat that this is a negative result, it could mean that some other proteins besides CP190 are in the complex.

While we were unable to generate a “native” Insv complex with bacterially expressed protein, we found that the protein–protein interaction domains play important roles in DNA binding. The Insv BEN domain is unable to bind to DNA in EMSAs in the absence of a dimerization (multimerization) domain, either an artificial dimerization domain like GST, or an ~ 40-amino acid sequence located just upstream of the BEN domain. At low concentrations, the C-BEN protein binds to the 32-bp probes (P3 and P1) containing the palindromic CCAATTGG sequence as a dimer; however, as input protein concentration is increased, the predominant shift corresponds to a C-BEN tetramer. Taken together with the crystal structure, this would suggest that a single C-BEN dimer interacts with the palindromic sequence, while the other C-BEN domain dimers in the tetramer could interact non-specifically with sequences elsewhere in this small probe, or instead might link together a second P3/P1 probe. For F-Insv, our experiments with bacterially expressed proteins indicate that it assembles into a mixture of mostly dimers and tetramers, and consequently, these two forms should be the predominant species bound to short P3 and P1 probes. However, the full-length protein is also capable of generating a series of high-order multimers with these short probes via protein–protein interactions.

Even more complicated shift patterns are observed for the two larger probes, P2 and 2xPal, and these likely reflect, at least in part, the multimerization properties of the Insv protein. For the C-BEN:P2 combination, a prominent shift is observed for tetramers, but not dimers. In contrast, dimers, tetramers, and even C-BEN hexamers are observed for the 2xPal probe. For both probes, higher-order multimers predominate as the concentration of the C-BEN protein is increased. This is also true for HT:F-Insv. Although HT:F-Insv binds to these probes as either a dimer or a tetramer at low concentrations, higher-order multimers are observed as the protein concentration is increased. Since these higher-order multimers are not readily detected in either gel filtration or cross-linking experiments, it seems likely that their formation by bacterially expressed proteins is stimulated by DNA binding.

Our studies indicate that the biochemical properties of Insv protein conform with those that might be expected for an architectural factor. Like dCTCF, Insv is a multivalent DNA-binding protein and thus could bridge distant DNA sequences. The C domain alone is sufficient to mediate tetramer formation, and this enables the C-BEN protein to bind to the extended P2 DNA probe even though it lacks a consensus CCAATTGG palindrome. The C-BEN protein can also assemble into higher-order complexes in the presence of suitable DNA substrates. However, this is not the only domain that contributes to multivalent DNA binding. The N-terminal coiled-coil domain is also able to generate tetramers on its own, while the full-length Insv protein can form higher-order multimers not only with extended DNA probes, but also with short probes containing just the palindrome.

In addition to being important for DNA binding in vitro, our rescue experiments indicate that self-interactions play a key role in the architectural functions of Insv in vivo. To determine if the N-terminal coiled-coil domain is important for Insv boundary function, we took advantage of a mutant *Fab*-*7* replacement, *Fab*-*7*^*GAGA1-5*^, which we used in a previous study to show that *insv* contributes to *Fab*-*7* boundary function. The boundary function of *Fab*-*7*^*GAGA1-5*^ is slightly impaired, and flies carrying this replacement exhibit weak GOF and LOF transformations in A6 (PS11); however, when combined with an *insv* null allele, boundary function is completely disrupted and the flies resemble a classical *Fab*-*7* deletion. We found that a genomic *insv* transgene fully rescues the effects of the *insv* null allele on the *Fab*-*7*^*GAGA1-5*^ boundary function, and the spectrum of weak GOF and LOF phenotypes in the rescued flies is the same as that observed for *Fab*-*7*^*GAGA1-5*^ in an otherwise wild-type background. Interestingly, a different result was obtained when we drove *insv*^+^ expression with a ubiquitously active *ubi* promoter. Instead of a combination of weak GOF and LOF phenotypes, the rescued *insv*^*23B*^; *ubi*-*insv*^+^ *Fab*-*7*^*GAGA1-5*^ flies are fully wild type. As noted in Results section, a likely explanation for this discrepancy is that the *ubi* promoter is active in cells or tissues that do not normally express the *insv* gene or do not express “sufficient” amounts of the Insv protein. In either case, the presence of “ectopic” Insv protein in these cells or tissues is able to compensate for the weak boundary defects induced by the mutations in the *Fab*-*7*^*GAGA1-5*^ replacement. While the *ubi*-*insv*^+^ transgene rescues *Fab*-*7*^*GAGA1-5*^ to wild type, ubiquitous expression of Insv does not compensate for the boundary defects of the *HS1 *+ *HS2* replacement. Instead, *insv*^*23B*^; *ubi*-*insv*^+^ *HS1 *+ *HS2* flies have the same set of phenotypic defects as *HS1 *+ *HS2* flies in a wild-type *insv* background.

In contrast to the wild-type Insv protein, the boundary function of a protein carrying a deletion for part of the N-terminal coiled-coil domain, Insv^d50*-*79^, is substantially impaired. One function of the coiled-coil domain is to promote protein stability. We found that the level of the Insv^d50*-*79^ protein expressed by the *ubi* transgene is substantially lower than the wild-type Insv protein. While maintaining sufficient levels of protein is clearly important, this does not appear to be the reason why the Insv^d50*-*79^ protein is unable to rescue the boundary defects in *insv*^*23B*^; *Fab*-*7*^*GAGA1-5*^ flies. We found that the Insv^d50*-*79^ protein had only a marginal effect on boundary function when we expressed it at the same level as the wild-type Insv protein. By contrast, the wild-type protein not only rescues the effects of the *insv*^*23B*^ mutation, but also compensates for the boundary defects of the *Fab*-*7*^*GAGA1-5*^ replacement. Thus, the effects of the d50-79 deletion on the architectural functions of Insv are likely underestimated in our experiments. In this context, it is interesting to note that the mammalian BEN domain proteins, Nac1 and Nac2, have BTB protein interaction domains [19]. BTB domains are known to mediate multimerization as well as heterologous protein–protein interactions. Taken together, our studies show that the full-length protein should be capable of linking distant sequences together in *cis* and in *trans*, either via bivalent tetramers, or via protein–protein interactions between tetramers bound to DNA target sequences.

This is not the only sort of protein–protein interactions that could support linkage of distant sequences in *cis* and *trans*. Insv is also capable of heterologous protein–protein interactions. We have shown that the N-terminal half of the Insv has a CP190 interaction domain. Since CP190 can bind to DNA (at least non-specifically), this should also stabilize Insv binding to its target sequences. While mutation of the CP190 interaction domain had only modest effects on *Fab*-*7* boundary activity, we did observe several CP190-dependent Insv binding sites in polytene chromosomes. This suggests that there are at least some chromosomal contexts in which Insv binding, and consequently, functions, that will depend upon its ability to associate with CP190. Additionally, it would not be surprising if Insv interacted with yet other proteins and/or is subject to post-translational modifications such as sumoylation that facilitate formation of complexes. Clearly, it will be of interest to isolate and characterize the endogenous Insv complex.

## Conclusions

Eukaryotic chromosomes are subdivided into discrete topological domains by special elements called boundaries or insulators. Critical to their architectural activities in flies is their ability to pair with each other. Pairing is mediated by direct physical interactions between the boundary elements. These pairing interactions are specific, between compatible boundaries, and are typically orientation dependent. Pairing between heterologous boundaries in the same chromosomal neighborhood occurs in *cis*, while pairing between homologous boundaries (self-pairing) occurs in *trans*. Two mechanisms for specific pairing interactions in flies have been identified. One is protein–protein interactions between factors associated with the paired boundaries, while the other is the deployment of multivalent DNA-binding complexes that can bind simultaneously to distant sequences. Here we have shown that the BEN domain protein Insv self-assembles into multivalent DNA-binding complexes and interacts with the boundary factor CP190. We also show that the N-terminal multimerization domain is critical for functioning of Insv in the BX-C boundary *Fab*-*7*.

## Materials and methods

### Insv expression transgenes

The genomic rescue construct, *insv*^+*3.67*^, contains a 3.67-kb fragment from the *insv* genomic region. It was amplified according to Duan et al. [22]. To express Insv protein variants in flies, we generated an expression vector, pUbi. The vector has a ubiquitin-63E (Ubi) promoter, a polylinker, and SV40 terminator, an *attB* site for the phiC31 integration system, and finally a *yellow* gene as a transformation marker. The coding sequence of Insv (FBgn0031434) and Insv d50-79, lacking part of the N-terminal coiled-coil domain, and Insv d135-150, deleted for the CP190 interaction domain, were amplified by PCR with primers containing restriction enzyme sites and cloned into pUbi vector. To generate the 5x-Pita-ubi-insv^d50*-*79^ construct, we modified the pUbi vector by inserting five Pita-binding sites upstream of the Ubi promoter [17]. All constructs were injected into preblastoderm embryos containing *attP* site at cytogenetic location 86F (y^1^M{vas-int.Dm}ZH-2A w*; M{3xP3-RFP.attP}ZH-86Fb; Bloomington stock RRID: BDSC_24749) [61].

### Fly stocks and genetic crosses

All flies were maintained at 25 °C on standard medium. The *Fab*-*7*^*GAGA1-5*^ and* HS1 *+ *HS2* replacements were generated using the *Fab*-*7attP50* landing platform described earlier [63]. The null mutation of *insv* gene, *insv*^*23B*^, was provided by Eric Lai (Department of Developmental Biology, Sloan-Kettering Institute, New York) [20, 21]. *Oregon*-*R* was used as wild type. In rescue experiments, transgenic lines with an expression construct on the third chromosome (*insv*^+*3.67*^ or the *ubi*-driven variants) were recombined with *Fab*-*7*^*GAGA1-5*^. All recombinants were verified by PCR and then introduced into an *insv*^*23B*^ mutant background.

### Cuticle preparations and immunostaining

Adult abdominal cuticles of homozygous enclosed 3–4-day-old flies were prepared essentially as described in Mihaly et al. [46] and mounted in Hoyer’s solution. Squashed salivary gland specimens were prepared and stained following standard protocols [59, 60]. Primary antibodies were rabbit polyclonal anti-Insv at 1:1000 dilution and rat polyclonal anti-CP190 at 1:1500 dilution. Secondary antibodies were goat anti-rabbit Alexa Fluor 555 and goat anti-rat Alexa Fluor 488 (Molecular Probes) used at 1:1000 dilution, and DNA was counterstained with DAPI. Stained polytene chromosomes were mounted in the Vectashield (Vector Labs) mounting medium and analyzed using a Nikon Eclipse Ti2 fluorescent microscope (objectives: Nikon Plan Apo 100 × 1,4 oil; camera: DS-Qi2; acquisition software: NIS-Elements BR 4.30.00; Japan). The images were processed using ImageJ software (version 1.51n) and Adobe Photoshop CS6.

### Bacterially expressed Insv proteins

DNA constructs for protein expression described above were cloned into the pGEX-5X2, pETSumo, and His6-Thioredoxin plasmid vectors. Starter cultures of *E. coli* cells transformed with these constructs were used to inoculate 1 L of LB broth containing ampicillin (50 μg/mL) or kanamycin (50 μg/mL). Cells were grown at 37 °C until the OD600 reached about 0.5 and then induced with 0.01 mM IPTG at 23 °C for 18 h. Cell pellets were frozen on dry ice and then thawed in a room-temperature water bath. Cell pellets for His6-Sumo-fused (HS) and His6-Thioredoxin-fused (HT) proteins were resuspended in lysis buffer (50 mM NaH_2_PO_4_, pH 8, 300 mM NaCl, 20 mM imidazole, 0.5% Triton X-100) with 1 mM dithiothreitol (DTT), 2 mM PMSF, 10 μg/mL DNase (Sigma), protease inhibitor cocktail tablets (Roche), and 1 mg/mL lysozyme (Sigma) and incubated on ice for 1 h. Cell pellets for GST-fused proteins were resuspended in 1x PBS, 0.5% Triton X-100 with protease inhibitor cocktail tablets (Roche), 1 mM DTT, 2 mM PMSF, 10 μg/mL DNase (Sigma), and 1 mg/mL lysozyme (Sigma) and incubated on ice for 1 h. Cells were lysed using Branson Analog Ultrasonic Processor Cell Disruptor (50% duty cycle for 1 min at 5 output control). The cell lysate was clarified by centrifugation at 15,000*g* and fractionated using Ni–NTA Agarose (Qiagen) for HS and HT fusion proteins and Glutathione-Agarose (Sigma) for GST fusion proteins. HS and HT fusion proteins were eluted using lysis buffer with the addition of 250 mM imidazole. GST fusion proteins were eluted using elution buffer (120 mM GSH, 300 mM NaCl, 100 mM Tris pH 8.5, 0.5% Triton X-100, 1 mM DTT, 1 mM EDTA, 10% glycerol). Protein concentrations were determined by SDS-PAGE analysis of experimental proteins, and bovine serum albumin (BSA) was used as a standard.

### Electrophoresis mobility shift assays (EMSAs)

In a total of 50 μL reaction, 1 pM of probe was 5’-end labeled with [γ-^32^P] ATP (MP Biomedicals) using T4 Polynucleotide Kinase (New England Biolabs). The reaction was incubated for 45 min at 37 °C. Labeled probe was separated from free ATP using columns packed with Sephadex G-50 fine gel (Amersham Biosciences). The eluted volume was adjusted to 100 μL using deionized water for a final concentration of 10 fM/μL labeled probe. For binding reactions, a 20-μL volume consisting of 25 mM Tris–HCl (pH 7.4), 100 mM KCl, 1 mM EDTA, 0.1 mM DTT, 0.1 mM PMSF, 0.03 mg/mL BSA, 10% glycerol, 0.5% Triton X-100, 0.25 mg/mL poly(dI-dC), 0.5 μL labeled probe, and 1 μL of protein or nuclear extracts. Nuclear extracts and in vitro translated proteins were prepared as in [51, 52]. In samples containing unlabeled competitor DNA, the DNA was included so that the final concentration of the competitor was at 50-100-fold excess. The reaction mixture described above was incubated for 30 min. at room temperature and loaded onto a 4% acrylamide:bis-acrylamide (1x TBE, 2.5% glycerol, and 0.5% Triton X-100) gel. The 36-64 lane gels were electrophoresed at 60 V for 2.5 h at 4 °C with a 0.5x TBE, 2.5% glycerol, and 0.5% Triton X-100 running buffer, and 20 lane gels were electrophoresed at 180 V for 3 h at 4 °C with the same running buffer. Gels were dried on 3MM chromatography paper (Whatman) and imaged using a Typhoon 9410 scanner and Image Gauge software and/or X-ray film

### Probes

- *P3:* CCACCGCAAAATCCAATTGGAAGAGAGCGACT
- *P3 Mutant:* CCACCGCAAAATCCCCGTGGAAGAGAGCGACT
- *P2#7:* CTTGCGCAGGACTTTTGAGATTCTATTAAATTCTAACAAGATTTCAAGCTGTGTGGCGGGGGGAAGAGGAAGAGAGCGGAAAGTGCAGCGC*CCAATAAG*CAAATGGCAGCTGTCACG
- *2X Pal:* CTCGAGGGGTTTCTTTCCCAATTGGAAATGCGTCCTGTCGAGGGGTTTCTTTCCCAATTGGAAATGCGTCCTGTCGACGGTATCGATAAGCTTGATATCGAATTCCTGCAGCCCGGGGATCC

### Size exclusion chromatography

For size estimations, 500 μL of the eluted protein was loaded onto Superdex 200 10/300 size exclusion column (GE Healthcare) or Superose 6 10/300 size exclusion column (GE Healthcare). Fractions of 500 μL were collected from 7 or 8 mL to 24 mL. Fractions were analyzed by SDS-PAGE to identify factions containing the protein of interest and with EMSAs to determine DNA-binding activity. Size exclusion standards (Bio-Rad) ranging from 1.35 to 670 kDa were used to calculate the partition coefficients and estimate the sizes of the protein complexes.

### Two-hybrid assay and in vitro interactions

Two-hybrid assays were carried out using the yeast strain pJ694A and plasmid vectors, pGBT9 and pGAD424, from Clontech (Palo Alto, CA). After transformation using the lithium acetate method, cells co-expressing fusion proteins were selected by growth on media lacking tryptophan and leucine. After 2 days of growth at 30 °C, the cells were plated on selective media without tryptophan, leucine, histidine, and adenine, and their growth was compared after 2–3 days. Each assay was repeated three times.

MBP-pull-downs were performed with Immobilized Amylose Agarose (New England Biolabs) in buffer C (20 mM HEPES–KOH, pH 7.7; 150 mM NaCl, 10 mM MgCl_2_, 0.1 mM ZnCl_2_, 0.1% NP40, 10% (w/w) glycerol). BL21 cells co-transformed with plasmids expressing MBP-fused Insv derivatives and 6xHis-fused CP190 derivatives were grown in LB media to an A600 of 1.0 at 37 °C and then induced with 1 mM IPTG at 18 °C overnight. Cells were disrupted by sonication, centrifuged, applied to resin for 10 min at + 4 °C; after that, resin was washed four times with buffer C containing 500 mM NaCl and elution performed with 50 mM reduced glutathione, 100 mM Tris, pH 8.0, 100 mM NaCl, for 15 min. 6xHis-pull-down was performed similarly to Co-IDA resin (Pierce) in buffer A and washed with buffer A containing 30 mM imidazole, and proteins were eluted with buffer B.

Chemical cross-linking with glutaraldehyde or EGS was carried out for 10 min at room temperature in PBS buffer containing 1 mM β-mercaptoethanol. Prior to cross-linking, protein concentration was adjusted to 10 μM. Cross-linking was quenched with 50 mM glycine, and samples were resolved using SDS-PAGE followed by silver staining.

### Antibodies

The primary antibodies used for immunofluorescence were as follows: Insv (aa 1–267) [62] and CP190 (aa 308–1096) raised in rabbits and rats, respectively, purified from the sera by ammonium sulfate fractionation, followed by affinity purification on CNBr-activated Sepharose (GE Healthcare), according to standard protocols. Other antibodies were anti-FLAG (M2, Sigma), and secondary antibodies conjugated to either Alexa Fluor 488 or Alexa Fluor 555 were purchased from Molecular Probes (Life Technologies).

### Chromatin immunoprecipitation experiments

The methods used for ChIP experiments are described in [62].

## Additional file

**Additional file 1: Fig. S1.** Fractionation of HS: F-Insv on a Superose 6 10/300 size exclusion column. **Fig. S2.** Spatial patterns of Insv expression in wild type and transgenic rescue lines. **Fig. S3.** Wild type and mutant Insv proteins are expressed at the same level. **Fig. S4.** ubi-insv^*d135 -150*^ rescues the insv^*23B*^; Fab-7^*GAGA1-5*^ combination.
