# Supplementary material for: Functional dissection of the developmentally restricted BEN domain chromatin boundary factor Insensitive
Source: Epigenetics Chromatin. 2019 Jan 3;12:2. doi: 10.1186/s13072-018-0249-2 (PMC6317261; doi:10.1186/s13072-018-0249-2)
Supplement: Supplementary file 1 — Additional file 1: Fig. S1. Fractionation of HS: F-Insv on a Superose 6 10/300 size exclusion column. Fig. S2. Spatial patterns of Insv expression in wild type and transgenic rescue lines. Fig. S3. Wild type and mutant Insv proteins are expressed at the same level. Fig. S4. ubi-insvd135 -150 rescues the insv23B; Fab-7GAGA1-5 combination. [file 13072_2018_249_MOESM1_ESM.doc]

**Supplemental Figures**


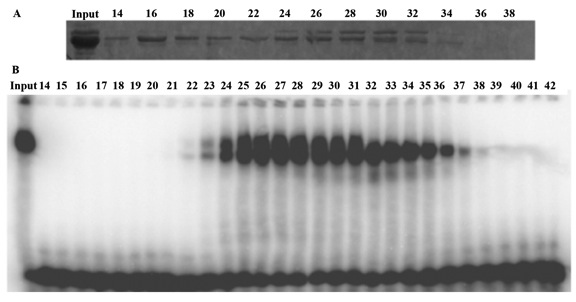


**Fig. S1: Fractionation of HS: F-Insv on a Superose 6 10/300 size exclusion column.**  Panel A. Fractions (as indicated) eluted from the Superose 6 10/300 size exclusion column were analyzed by SDS polyacrylamide gel electrophoresis and the gel stained with commassie blue. Insv protein is detected in fractions that elute early from the column. Panel B. Fractions (as indicated) eluted from the size exclusion column were tested for DNA binding activity using the P3 probe. DNA binding activity elutes over a broad range of predicted molecular weights from ~420 kDa-120 kDa. Protein eluting in the early fractions does not generate a shift with the P3 probe.


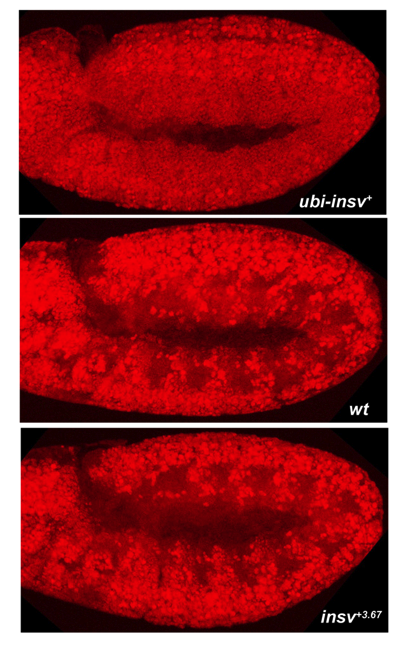


**Fig. S2: Spatial patterns of Insv expression in wild type and transgenic rescue lines.**  *Drosophila* embryos at stage 11, just before germband retraction. Expression pattern of Insv in wild type (wt) and in the *insv^23B^* mutants rescued by the genomic transgene *insv^+3.67^* or by the *ubi-insv^+^*. Expression of Insv in wild type and in *insv^+3.67^* are very similar. By contrast, Insv is more broadly expressed in embryos carrying the *ubi-insv^+^* transgene.

**
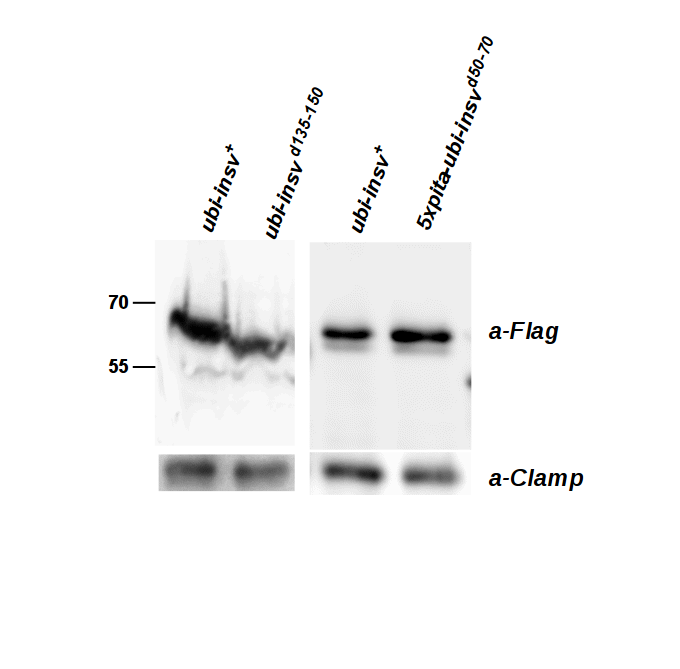
**

**Fig. S3: Wild type and mutant Insv proteins are expressed at the same level.** Western blot analysis of total protein extracts prepared from homozygous adult flies of *ubi-insv^+^*, *5xpita ubi-insv^d50-70^*, and *ubi-insv^d135 -150^* lines*.*  Insv protein was detected with an anti-FLAG antibody. Antibody recognizing the Clamp protein was used as the loading control.


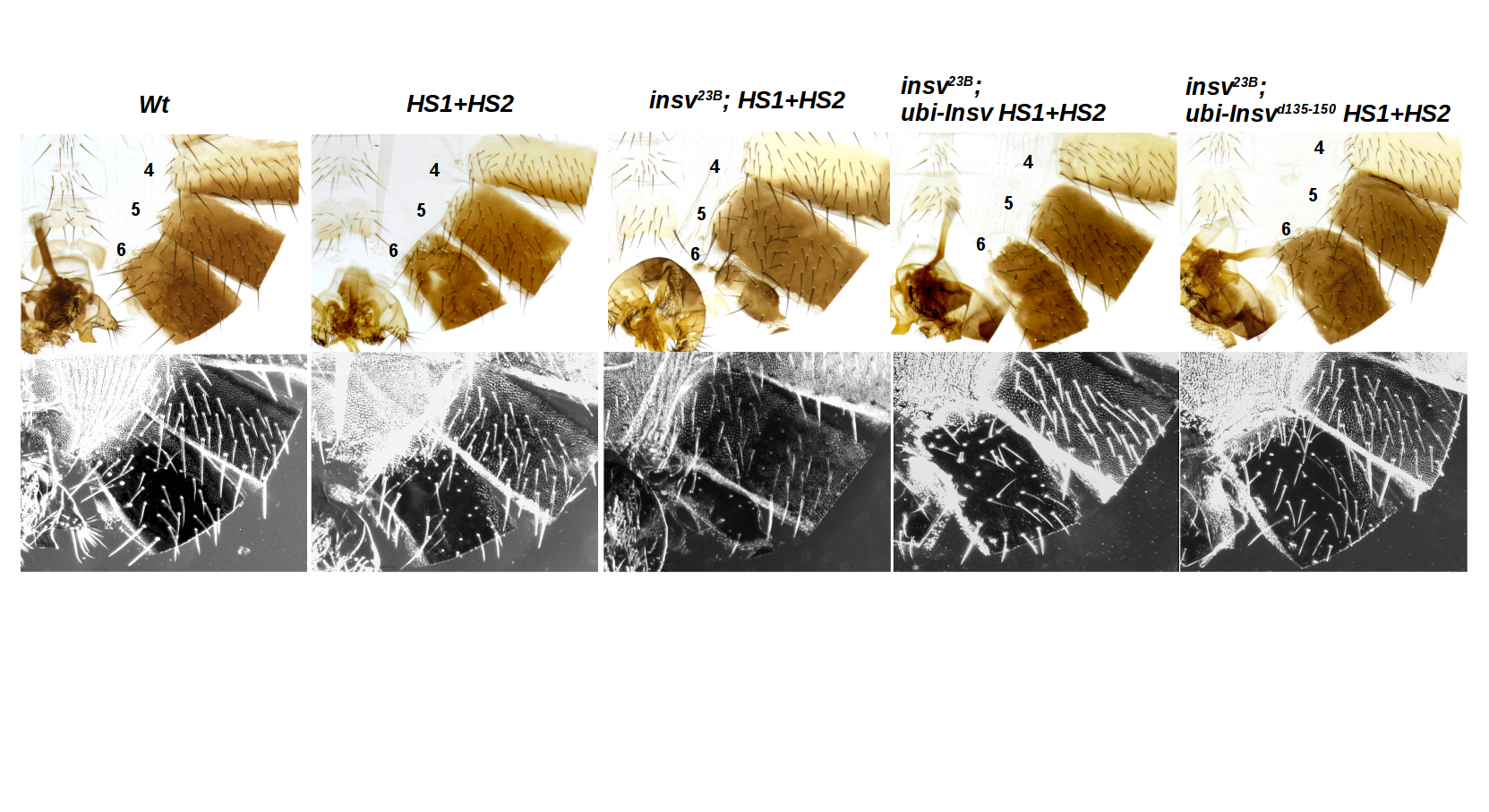


**Fig. S4: *ubi-insv^d135 -150^* rescues the *insv^23B^; Fab-7^GAGA1-5^* combination.** Panel A: Adult abdominal cuticle preparations of a wild type male. The 5th and 6th tergites are pigmented, the A6 sternite is recognizable by the absence of bristles and a specific form. Trichomes are visible in the dark field and cover all the surface of the A5 tergite and only a thin stripe along the anterior and ventral edges of the A6 tergite. Panel B: Male fly with the *HS1+HS2* replacement. The *HS1+HS2* replacement displays tissue specific defects in boundary function (Kyrchanova *et al.* 2017). The A6 sternite is missing. This phenotype arises from a GOF transformation of A6 (PS11) into a duplicate copy of A7 (PS12). Though boundary function is disrupted in the ventral cuticle, it is largely retained in the dorsal tergite. In most *HS1+HS2* male flies, the tergite is nearly the same size as in wild type. Moreover, judging from the characteristic trichome pattern, the A6 tergite is also properly specified. Panel C: *insv^23B^*; *HS1+HS2* males. In the absence of the endogenous Insv, *HS1+HS2* males show a strong GOF transformation of A6 to A7 as revealed by the marked reduction of the A6 tergite and the absence of the sternite. Panel D. *ubi-insv^+^* transgene rescues the boundary defects of *insv^23B^*; *HS1+HS2*. Flies resemble *HS1+HS2* flies. Panel E. The *ubi-insv^d135 -150^* transgene rescues the boundary defects of *insv^23B^*; *HS1+HS2*. Flies resemble *HS1+HS2* flies.
